# Validity and Reliability of the Short Physical Performance Battery Tool in Institutionalized Spanish Older Adults

**DOI:** 10.3390/nursrep13040114

**Published:** 2023-09-30

**Authors:** Mirian Santamaría-Peláez, Jerónimo J. González-Bernal, Álvaro Da Silva-González, Elena Medina-Pascual, Ana Gentil-Gutiérrez, Jessica Fernández-Solana, Juan Mielgo-Ayuso, Josefa González-Santos

**Affiliations:** 1 Department of Health Sciences, University of Burgos, 09001 Burgos, Spain; mspelaez@ubu.es (M.S.-P.); jejavier@ubu.es (J.J.G.-B.); ada@ubu.es (Á.D.S.-G.); jfmielgo@ubu.es (J.M.-A.); mjgonzalez@ubu.es (J.G.-S.); 2 Medical Services of Nursing Home, Diputación Provincial, 09001 Burgos, Spain; 3 Burgos University Hospital, 09006 Burgos, Spain; emedinap@saludcastillayleon.es

**Keywords:** short physical performance battery, comprehensive geriatric assessment, older adults, institutionalization, psychometric properties

## Abstract

Background: In order to be used safely, accurately and reliably, measuring instruments in the health field must first be validated, for which the study of their psychometric properties is necessary. The Short Physical Performance Battery (SPPB) tool is a widely used clinical assessment test that has been approved for usage across several nations, languages and demographics. Finding SPPB’s psychometric properties for a sample of institutionalized older individuals is the aim of this research. Methods: This is a multicenter, retrospective and observational study of the psychometric properties of the Short Physical Performance Battery tool with a convenience sample of 194 institutionalized older adults. Reliability (internal consistency) and validity (construct validity and convergent validity) tests were performed. Results: The results show a very good internal consistency, construct validity and convergent validity. In addition, the factorial structure of the SPPB is provided, which reflects that it is a unidimensional scale. Conclusions: In conclusion, the Short Physical Performance Battery is a valid and reliable tool for use with institutionalized older adults. Its use is recommended as part of the Comprehensive Geriatric Assessment for the evaluation of the physical or functional sphere. This study was not registered.

## 1. Introduction

Comprehensive Geriatric Assessment (CGA) is a widely used tool that enables the detection of risk factors and provides a comprehensive assessment of older people in an integrated manner. It consists of four main sections: clinical, physical, mental and social. Different and varied evaluation tools are used to provide a reliable measure of all parameters in a common language [1].

Among the data that are normally collected by the professionals of the elderly care centers through the CGA, and framed in the functional section, those referring to physical functioning stand out. The physical performance of older people is closely related to the concepts of frailty, comorbidity and sarcopenia. Physical performance tests are strongly associated with the onset of functional dependence; therefore, their use is advised to develop a risk assessment strategy that could identify subgroups of older people, independent in activities of daily living (ADL), who are at higher risk of functional dependence [2].

Functional health is defined as the ability to conduct the tasks and activities that are important in the daily life of individuals. In contrast, functional decline is a common manifestation in numerous adverse medical conditions, usually originating from acute and chronic problems that synergistically impair independence in performing both ADL and the instrumental activities of daily living (IADL) [3].

The assessment of functional health can serve as an early warning system, as it may indicate the onset of health problems in older adults while also enabling the development of personalized care plans to address specific deficits or limitations [4]. Thus, measuring it can impact healthcare policies and cost estimates for older adults and is also crucial in clinical research involving older adults because it provides reference data and helps to evaluate the effectiveness of interventions aimed at improving or maintaining functional independence [5].

For all this, measuring functional health in older adults has significant implications for healthcare delivery, resource allocation, research and the overall well-being of older populations. These implications underscore the importance of accurate and comprehensive functional assessments in both clinical practice and public health initiatives.

Within the guidelines of the European Innovation Partnership on aging, the prevention and early diagnosis of functional and cognitive impairment with interventions aimed at frailty is defined as a main line. In addition, the Framework Programme for Research and Innovation 2014–2020 (Horizon 2020) included six sub-programs. The Innovative Medicines Initiative 2013 also planned the “development of innovative therapeutic interventions for physical frailty and sarcopenia, as a prototype geriatric indication” [6].

In the 1990s, the World Health Organization referred to active aging, which is defined as “the process of optimizing opportunities for health, participation and security with the aim of improving quality of life as people age” [6]. Thus, maintaining autonomy and independence through the years are the primary objectives.

The Consensus Document on Frailty and Falls in Older People establishes inactivity as the most relevant frailty risk factor [6] and proposes the Short Physical Performance Battery (SPPB) instrument as a screening tool for frailty and fall risk among older people.

Older adults who are frail may progress toward dependence and disability; this process follows a pattern beginning with the impairment of mobility and flexibility, which subsequently progresses to difficulties in performing IADL and eventually prevents the proper performance of ADL.

Several simple tests of physical performance are strongly associated with the occurrence of functional dependence. These results support the potential use of physical performance tests to develop a risk assessment strategy that could identify subgroups of older people, independent in all ADL, who are at an increased risk of functional dependence [2]. 

Other studies have confirmed its usefulness as a screening tool to detect the frailty syndrome in community-dwelling older adults [7,8,9,10]. However, a systematic review [11] determined that, although it is a reliable and valid tool for physical performance in older adults over 60 years old, it has a limited scope and is more appropriate for frail older adults who can walk and are cognitively capable of following instructions. Additionally, it is not particularly sensitive to change, so it is a useful instant screening tool, but its usefulness is limited in long-term follow-ups [11].

The measurement of health-related aspects becomes a fundamental tool for evaluating health outcomes and guiding clinical decisions. However, for these tools to be effective, it is essential to confirm their reliability and validity through appropriate psychometric tests. Quantifying these aspects involves first clearly defining them and then developing items and scales that represent them. Unlike objective measurements, such as laboratory tests, the measurements of complex and/or subjective constructs often lack established psychometric properties, necessitating expertise in their development and interpretation. To obtain accurate inferences from these measurements, it is crucial that the instruments used undergo a robust process of development and psychometric testing to assess their validity and reliability, especially when measuring “subjective” or complex phenomena and when controlling for known errors. Without reliable and valid measurements of health-related constructs, the utility of research findings is compromised and its use can lead to incorrect diagnoses in patient safety issues and the implementation of inappropriate preventive strategies. One of the most common challenges in health sciences research is the validation and reliability of measurement instruments [12].

The psychometric characteristics of the SPPB scale have been studied in different places and populations, obtaining good results. There is scientific evidence of its validity [13,14]; reference values were established according to gender and three age groups (between 70 and 75, between 76 and 80, and over 80). The SPPB proved to be a valid and reliable tool in the assessment of physical fitness in Colombian older adults [15,16]; Norwegians [17,18], Brazilians [10] and Canadians [19]; in different pathologies, such as cardiac [14,20], asthma [21], chronic obstructive pulmonary disease [22], chronic kidney disease [23] and multiple sclerosis [24]; and in different contexts, especially in the hospital environment [14,25] and community [7,8]. However, studies in institutionalized older adults are not as common [26].

The SPPB scale has been found to be a useful tool for the assessment of lower extremity function in older adults and is a good predictor of numerous health outcomes, such as ADL dependence, mobility difficulties, disability, hospitalization, prolonged hospitalization, institutionalization and even death, as well as a poorer quality of life [14,19,27,28,29].

A systematic review on performance-based physical function assessment in people living in a community concluded that the SPPB is the most recommended tool in terms of validity, reliability and responsiveness [30]. Another review [31] showed that Gait Speed or SPPB were the most valid, reliable and feasible tools for the assessment of physical performance in a home environment.

Despite these previous studies, the psychometric properties of the SPPB in institutionalized Spanish older adults has not been previously explored, which is the main objective of this study. In addition, three specific objectives for this research are established: (1) to determine the reliability of the SPPB scale; (2) to determine the construct validity of the SPPB scale; and (3) to determine the convergent validity of the SPPB scale.

## 2. Materials and Methods

### 2.1. Participants

This research was a retrospective observational study, for which a total of 202 entries were recorded in a dossier prepared for this purpose and distributed to the participating centers, of which 8 were discarded because they were incorrectly completed or incomplete. Thus, the sample consisted of a total of 194 institutionalized older adult people in four residential centers: Burgos (n = 63); Aranda de Duero (n = 38); Salamanca (n = 76); and San Sebastián de los Reyes (n = 17).

The geographical distribution of the sample is as follows:

- Province of Burgos (Spain): 63 in Burgos city and 38 in Aranda de Duero.
- Province of Salamanca: 76 in Salamanca city.
- Province of Madrid: 17 in San Sebastián de los Reyes.

In terms of the type of center, all were residential. The ownership varied as follows: 177 participants were in privately and 38 in publicly owned centers.

The ages of the participants were between 63 and 97 years.

### 2.2. Data Collection

In order to obtain the sample, several centers managed by Grupo Norte were contacted. This is a business group dedicated to the management of care services for the elderly, among other activities. After signing a collaboration and confidentiality agreement document with the participating centers, the data collection necessary for this research was conducted. The Ethics Committee of the University of Burgos positively assessed the research plan in the IR 11/2018 Approval Committee. 

Thanks to the professionals of the multidisciplinary teams, each of the participating centers performed the data collection as part of their routine documentation (each of these centers has its own approved data protection procedure in place, accordingly to legal requirements), and it was sent to the investigating team after a process of anonymization; from this point on, they were always treated anonymously and in aggregate. All study subjects participated voluntarily.

An anonymization procedure consisting of the following steps was established: First, data collection; second, coding; third, introduction of the data into the statistical program; fourth, data processing in the cross-sectional phase; and fifth, custody of the anonymized data.

Therefore, non-probability and convenience sampling was performed, during which no randomization procedure was conducted; this type of sampling is widely used in the health and social sciences. No sample calculation was performed and sampling errors were not taken into account.

The IBM SPSS-v5 (Statistical Package for the Social Sciences) software program was used for the statistical analysis.

### 2.3. Instruments

#### 2.3.1. Short Physical Performance Battery (SPPB or Guralnik Test)

It is a widely used performance test in geriatric medicine that has been validated in different study populations and is adjusted for age, gender and comorbidity. It is easy to use and does not require any equipment. The scale makes it possible to monitor follow-up over time and the evolution of the person; changes in 1 point in the SPPB are clinically significant [6], although there are studies that claim that it is not particularly sensitive to changes and, therefore, its usefulness is limited in long-term tracking [11]. It is a useful tool for the assessment of mobility limitations [6].

This tool is divided into three sections: balance (0–4 points): in the standing, semi-tandem and tandem positions; walking speed (0–4 points) in 2.4 or 4 m; and getting up from and sitting down on a chair five times (0–4 points). The established sequence must be respected and the administration time is between 6 and 10 min.

As normative values, scores can be between 0 and 12. Therefore, 0 is the worst situation and scores below 10 indicate poor physical condition, frailty and high risk of falls [6]. The ViviFrail multicomponent physical training program for the prevention of frailty and falls in the population over 70 years of age also proposes the following cut-off points [32]: severe limitation (dependent or disabled), SPPB 0–3; moderate limitation (frail), SPPB 4–6; mild limitation (prefrail), SPPB 7–9; and minimal limitation (autonomous or robust), SPPB 10–12.

#### 2.3.2. Barthel Index

It assesses and monitors progress in independence in self-care in patients with neuromuscular and/or musculoskeletal pathologies admitted to chronic hospitals [33].

The British Geriatrics Society recommends its use for the assessment of basic ADL in older patients and it is especially useful in rehabilitation units. It is administered in 5 min through the direct observation and/or questioning of the person or their caregivers. It assesses ten basic activities, and its total score ranges from 0 to 100 points (90 for wheelchair users). It has a very good reproducibility with weighted kappa correlation coefficients of 0.98 intra-observer and higher than 0.88 interobserver [33]. It has an excellent internal consistency with a Cronbach’s alpha of 0.90–0.92 [34]. It has cut-off points established in [3]: independence: 100; low dependence: 91–99; moderate dependence: 61–90; severe dependence: 21–60; and total dependence: <21.

#### 2.3.3. Lawton and Brody Scale

Developed for the older population, institutionalized or not, it assesses physical autonomy and IADL and is frequently used. It assesses eight instrumental activities. It is a hetero-administered questionnaire in which the person or their carers are consulted, with an administration time of 5–10 min [6].

It can be used for the assessment of the functional capacity of any person. Each area is scored according to the description that best corresponds to the subject, so that each area has a maximum of 1 point and a minimum of 0 points. The total score ranges between 0 and 8 points, where the lowest score implies a greater dependence.

It was translated, adapted and validated in Spanish, obtaining a high inter- and intra-observer reproducibility coefficient (0.94) [33] and a good inter-observer reliability coefficient, although it presents some problems of construct [6].

#### 2.3.4. Global Deterioration Scale and Functional Assessment Staging (GDS-FAST)

It is an easy-to-use, standardized tool that specifies the stage of clinical evolution of a patient, reflecting the changes in their functional state. It is considered to be a “generalisable and widely applicable global measure for the assessment of cognitive impairment secondary to primary degenerative dementia” [35]. It is widely used and is one of the most common classifications for the stages of Alzheimer’s disease [36]. It consists of seven degrees of impairment (GDS 1–GDS 7), in which both cognitive symptoms and functional impairments are assessed, where the highest level means a worse functional condition; it is the functional part that is of use in this research. At GDS 4, there is a deterioration of cognitive skills and, functionally, the ability to perform daily activities is affected. From GDS 5 onwards, the person being assessed can no longer survive without assistance, i.e., he/she would be dependent for basic ADL. The last two stages are further subdivided (SDG 6a–SDG 6e and SDG 7a–SDG 7f) [36] to explain the deterioration in each of them in more detail.

#### 2.3.5. Downton Risk Fall Index

This scale consists of 11 items and is intended to measure the risk of falling. Each item can be scored as 1 or 0. A total score of 3 or more is indicative of a high risk of falling. It is considered to have a good content validity and is a useful instrument for the prediction of fall risk in the residential setting [37].

The Downton scale was developed for older adults in intensive care units. Subsequently, research was conducted in residential facilities for the elderly [37], which concluded that it is also a useful instrument for the prediction of fall risk in the residential setting. This study also showed a higher sensitivity at three months.

### 2.4. Statistical Analysis

First, a descriptive analysis of the sample was performed. After this, a normality analysis was performed for the quantitative variables with the Kolmogorof–Smirnof test, the results of which show that the sample did not conform to normality (*p* > 0.05).

Subsequently, we proceeded to the psychometric analysis of the SPPB scale, for which several actions were performed. First, for the reliability analysis, internal consistency was tested by means of Cronbach’s alpha, correlations between the items and the total score, and the half-and-half test. For the validity analysis, construct validity was tested by means of an exploratory factor analysis, multi-dimensional scaling and the validity of known groups, comparing groups with and without fall risk as measured by the Downton Risk Fall Index. The convergent validity was analyzed by means of the correlation of the Barthel index, Lawton and Brody scale and GDS-FAST.

## 3. Results

The sample of 194 institutionalized older adult people had a mean age of 86.46 years (SD ± 9.01), with a maximum of 97 and a minimum of 63 years. Most of the participants were in a situation of dependency (46%) or frailty (43.8%); the rest were independent (10.2%) The average score obtained in the SPPB was 4.17; the group with the highest average was that of independent individuals (10.89) and, thereafter, it decreased as one moved towards dependency (0.99 on average).

In terms of gender distribution, 73.6% were women and 26.4% were men. The majority of the participants’ marital status was widowed, at 65%; 17.3% were single and 16.2% were married; the lowest percentage, that of divorced or separated individuals, was 1.5%. The average number of children was 1.76, with a maximum of 8 and a minimum of 0.

A total of 56.3% of the participants had primary education and 18.8% had no education at all, which is a similar percentage to that of those with secondary education (18.3%); only 2.5% had a university education. 

Regarding the main job performed, 37.6% were engaged in domestic activities; the rest of the jobs, divided by sector, were: 26.4% in the primary sector, 11.2% in the secondary sector and 23.9% in the tertiary sector. Appendix A shows the descriptive data of the quantitative variables.

### 3.1. Results of the Reliability Analysis

#### Results of the Internal Consistency

- Results of the Cronbach’s alpha

The obtained Cronbach’s alpha was 0.86. Additionally, the Cronbach’s alpha for each of the items that were eliminated ranged from 0.77 to 0.85, and the overall corrected item correlation was always higher than 0.69.

- Correlations items: total score

High correlations among all items of SPPB were found, with correlation coefficients ranging between 0.704 and 0.771 (*p* < 0.001). Correlations between each item and the total score of SPPB were also high, with correlation coefficients ranging between 0.839 and 0.940 (*p* < 0.001).

- Half-and-Half Test

The reliability of the SPPB scale was also examined using the half-and-half test as shown in Table 1—The Spearman–Brown coefficient obtained by means of the half-and-half test confirmed the good internal consistency of the SPPB, with a score of over 0.80.

### 3.2. Results of the Validity Analysis

#### 3.2.1. Results of the Construct Validity

- Exploratory factor analysis

A principal components analysis with oblique rotation was performed as the correlations between the items were higher than 0.70 in all cases. None of the items (speed, balance and get up) was eliminated, as they were all grouped into a single factor with factor loadings between 0.858 and 0.905.

In the proposed solution, eigenvalues greater than 1 determined that the scale would be composed of a single (unidimensional) dimension. This explains the 78.83% of variance; the three items had factor loadings higher than 0.80 within the single factor and communalities higher than 0.73 (Table 2, Table 3 and Table 4). Since it is a one-dimensional scale, rotation is not conducted.

Finally, a scale with a single dimension composed of three items was obtained. Bartlett’s test of sphericity was significant (282.48; gl = 3; *p* < 0.001) and the Kaiser–Meyer–Olkin sample size adequacy indicator was appropriate (0.726).

- Multidimensional scaling

**Figure 1:**
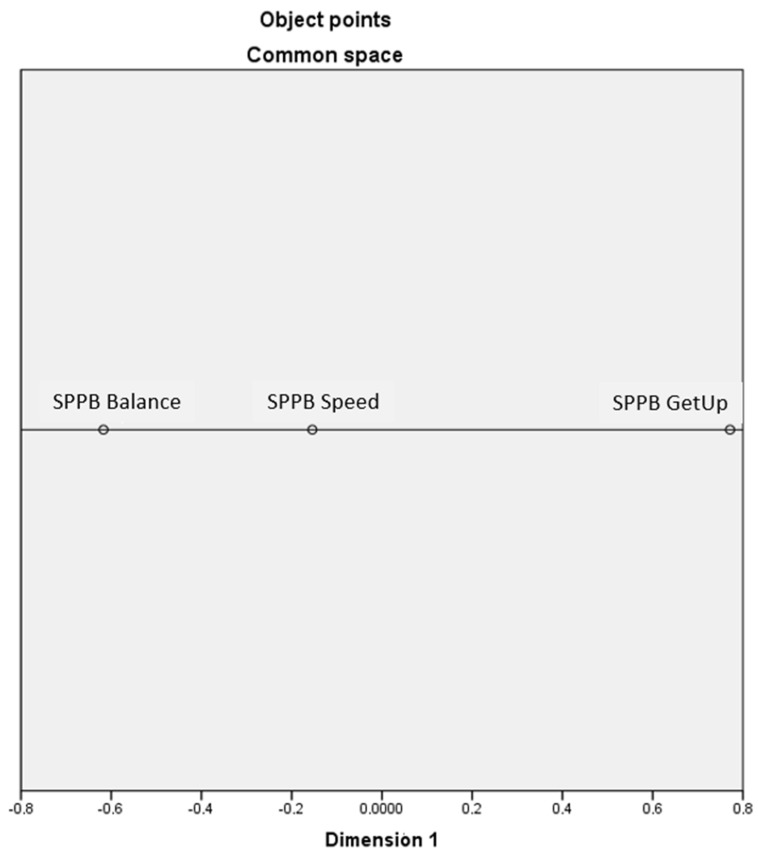
Multidimensional scaling graph.

The graph obtained from the multidimensional scaling analysis (Figure 1) shows that the SPPB is a one-dimensional scale; its final coordinates and distances are shown in Table 5 and Table 6.

The stress and fit measures show a very low stress index; so, the proposed model was considered appropriate (Table 7).

- Validity of the known groups

There is evidence associating a higher risk of falls with a worse physical performance [38,39], which is why the falls risk variable was chosen for this analysis. As a total score of 3 or more in the Downton index is indicative of a high risk of falling, the sample was divided in those who are at risk of falling and those who are not; these were the comparison groups used to perform this analysis.

Table 8 shows the existence of statistically significant differences in the SPPB score for the groups with and without fall risks.

#### 3.2.2. Results of the Convergent Validity

The scores of each SPPB item and the scale’s total score show a significant correlation with the ADL (Barthel Index), IADL (Lawton and Brody scale) and functionality (GDS-FAST) scores, as shown in Table 9.

## 4. Discussion

The descriptive statistics reveal that the study population is older people and that most of the participants are in a situation of dependency (46%) or frailty (43.8%), which is consistent with the data reported by other studies [6,40]. The sample was, therefore, considered to be in line with the situation in residential care homes for older people and representative of this population.

This study showed a Cronbach’s alpha coefficient of 0.863, which implies a good internal consistency of the scale, if we take into account that alpha values between 0.7 and 0.8 are considered “good” [41]. In turn, it is also necessary to point out that a value above 0.90 indicates that several items measure exactly the same thing (redundancy or duplication) [41]; so, we can consider that this is not the case with our scale. Taking also into account alpha values between 0.774 and 0.851 with each deleted item and that the total correlation with the corrected items is greater than 0.69, we can also affirm that it is not necessary to delete any of the items that make up the scale.

The results obtained for the item–total score correlation show good homogeneity, i.e., the three items are part of a single construct [41]. Subsequently, in our analysis, this was confirmed by the results obtained in the factor analysis used for construct validity.

On the other hand, the internal consistency assessed by means of the half-and-half test had a Spearman–Brown coefficient value greater than 0.80, which means, consistently with the rest of the results, a good internal consistency.

Based on the above, it can be suggested that the SPPB scale has adequate internal consistency; all its items are part of and measure the same construct and the linear relationship between the sum of the scores of the items with the measured construct is fulfilled [41].

In terms of validity, we decided to assess the construct validity and contingent validity, but content validity was not assessed, although quantitative tools, whose purpose is to collect information on the importance of a variable, are needed to verify the content validity through an analysis of the concept expressed in the variable [42].

The most commonly used content validation processes involve the assessment of the scale items by a panel of experts, but, in this case, it is an instrument whose use has been recommended by the Consensus Document on Frailty and Falls [6], which is widely used by geriatric physicians [43] and, therefore, assigns it de facto expert opinion. In addition, it is an instrument whose translation into Spanish has been used in other validation processes of its properties [13,15,27]; therefore, it is considered that this content validity process has already been conducted for this version of the tool.

As for construct validity, the exploratory factor analysis corroborated the version of the SPPB used in the literature and showed that it is composed of a single factor since the three items showed a correct theoretical grouping with this single factor. Bartlett’s test showed a good correlation between the variables (*p* < 0.001), and the Kaiser–Meyer–Olkin sample size indicator also obtained an optimal result above 0.7, as stated by Carvajal et al. [42] (2011), Sánchez-Martínez et al. [44] (2019) and Garmendia [45].

The literature proposes several ways to determine the unidimensionality of an instrument, most of them using the variance explained by the first factor extracted. Thus, it has been established that the amount of variance explained by the first factor should be, for some authors, higher than 20%, 30% or even 40% [46]; however, there is no consensus. The present research met this criterion in all cases, with a total variance explained by the first factor of 78.83%. However, due to this variability of criteria among authors, we proceeded to conduct multidimensional scaling, whose data corroborated that the SPPB is a unidimensional tool composed of three items.

In addition, to test the construct validity, we also examined the existence of differences between the two known subgroups, with and without risk of falls, because there are numerous studies that relate them. We used the cut-off point proposed by the Downton scale of risk of falls, which indicates that scores equal to or greater than 3 are indicative of a high risk of falls [37]. The results show that there are significant statistical differences between the groups of people with and without risk of falls, both for the SPPB total score and for each of its items, results in concordance with those of other studies that relate falls and/or fall risk to the SPPB [39,47,48,49] and, even that propose the SPPB as a good instrument in itself to measure the risk of falling [50] and to predict falls [6].

The total score of the SPPB and each of its component items also have positive correlations with the scores of the Barthel Index and Lawton and Brody scale and negative correlations with the score of the GDS-FAST. This shows that the worse a person’s physical performance is, the more limitations they have in ADL and IADL as well as overall impairment, and that the scores from the various scales tend to converge in the same direction. The findings show that the SPPB has strong convergent validity for the sample, as this link between the SPPB and ADL has also been confirmed in previous research [51,52,53].

The paper’s multicenter design and focus on a population with particular needs and features that call for proven evaluation tools should be acknowledged as positives. A blinding technique is indicated by the method by which the individuals in responsibility of data collection are distinct from those in charge of the statistical analysis of the data. The findings provide extremely valuable information that enables us to suggest the use of the SPPB to evaluate the physical capabilities of institutionalized older people; its use could be advised as a component of the CGA for the evaluation of its functional sphere, and it may also be a helpful marker for the beginning of frailty, dependence and fall risk. This would help in the clinical practice to identify and treat the most vulnerable people at an early stage.

It is important to emphasize the study’s shortcomings, which include the convenience sample it used, the absence of sample calculation or randomization and the fact that other indicators, such as fragility, were not used. To corroborate, contrast and amplify these findings, more research along similar lines is needed; such research should include more indicators to conduct comparative studies. Because studies are contradictory concerning the sensitivity to change and the usefulness of the scale for monitoring long-term changes [6,11], there is also a need to develop longitudinal lines of research that address this aspect.

## 5. Conclusions

The SPPB’s Spanish version is a reliable and valid resource for geriatric specialists working in these types of facilities since it has a strong validity and reliability for the assessment of the physical performance in institutionalized older adults.

The instrument was found to have a good internal consistency as a sign of its reliability. The SPPB also exhibits a strong convergent validity and strong construct validity for a unidimensional model.

The SPPB tool is advised for use as a component of the CGA for the evaluation of the physical or functional sphere, and it may also be a helpful marker for the beginning of frailty, dependence and fall risk.

## Figures and Tables

**Table 1:** Reliability statistics obtained with the half-and-half method.

Cronbach’s Alpha	Part 1	Value	0.851
		N of elements	2 ^a^
	Part 2	Value	1.000
		N of elements	1 ^b^
	N total of elements		3
Correlation between forms			0.694
Spearman–Brown Coefficient	Equal Length		0.819
	Unequal Length		0.833
Guttman split-half coefficient			0.701

^a^ The elements are: SPPB Balance and SPPB Speed. ^b^ The elements are: SPPB Speed and SPPB GetUp.

**Table 2:** Results of the exploratory factor analysis (total variance explained).

	Initial Eigenvalues			Sum of Squared Extraction of Variance		
Component	Total	% of Variance	% Cumulative	Total	% of Variance	% Cumulative
1	2.365	78.832	78.832	2.365	78.832	78.832
2	0.383	12.781	91.613			
3	0.252	8.387	100.000			

Extraction method: principal component analysis.

**Table 3:** Exploratory factor analysis: component matrix.

	Component 1
SPPB Speed	0.905
SPPB Balance	0.899
SPPB GetUp	0.858

SPPB: Short Physical Performance Battery. Extraction method: analysis of principal components.

**Table 4:** Exploratory factor analysis: communalities.

	Initial	Extraction
SPPB Speed	1.000	0.809
SPPB Balance	1.000	0.820
SPPB GetUp	1.000	0.736

Extraction method: analysis of principal components. SPPB: Short Physical Performance Battery.

**Table 5:** Multidimensional scaling: final coordinates.

	Dimension	
	1	2
SPPB Speed	−0.617	0.000
SPPB Balance	−0.154	0.000
SPPB GetUp	−0.772	0.000

SPPB: Short Physical Performance Battery.

**Table 6:** Multidimensional scaling: matrix of distances.

	SPPB Speed	SPPB Balance	SPPB GetUp
SPPB Speed	0.000		
SPPB Balance	0.463	0.000	
SPPB GetUp	1.389	0.926	0.000

SPPB: Short Physical Performance Battery.

**Table 7:** Multidimensional scaling: stress and fit measures.

Normalized raw stress	0.00000
Stress-I	0.00000 ^a^
Stress-II	0.00000 ^a^
S-Stress	0.00000 ^a^
Dispersion counted for (D.A.F.)	1.00000
Tucker’s congruence coefficient	1.00000

PROXCAL minimized the normalized raw stress. ^a^ Optimal scaling factor = 1.000.

**Table 8:** Comparison between the known groups. Mann–Whitney U test.

Variable	Median with Risk of Falling	Median without Risk of Falling	Mann–Whitney U	Z	*p*-Value
SPPB Balance	1	3	2058.00	−6.73	<0.001
SPPB Speed	1	2	2145.00	−6.49	<0.001
SPPB GetUp	0	1	2379.00	−6.34	<0.001
SPPB Total	2	6	1880.50	−7.10	<0.001

SPPB: Short Physical Performance Battery.

**Table 9:** Spearman’s correlation: SPPB with Barthel Index; Lawton and Brody scale; and GDS-FAST.

		Barthel	Lawton and Brody	GDS_FAST
SPPB Balance	Correlation coefficient	0.794 **	0.298 **	−0.474 **
	Sig. (bilateral)	<0.001	<0.001	<0.001
	N	194	194	194
SPPB Speed	Correlation coefficient	0.781 **	0.384 **	−0.472 **
	Sig. (bilateral)	<0.001	<0.001	<0.001
	N	194	194	194
SPPB GetUp	Correlation coefficient	0.760 **	0.399 **	−0.452 **
	Sig. (bilateral)	<0.001	<0.001	<0.001
	N	194	194	194
SPPB Total	Correlation coefficient	0.853 **	0.386 **	−0.516 **
	Sig. (bilateral	<0.001	<0.001	<0.001
	N	194	194	194

GDS_FAST: Global Deterioration Scale and Functional Assessment Staging; SPPB: Short Physical Performance Battery. ** Correlation is significant at the 0.01 level (bilat.).

## Data Availability

The data presented in this study are available on request from the corresponding author. The data are not publicly available due to due to agreement between the company and the principal investigator.

## Public Involvement Statement

Aim: The participants were included in the research with the objective of evaluating the psychometric properties of the scale. Methods: They participated voluntarily, and the purpose and procedure of the study and their involvement in it were explained to them. The sample used was convenience, as it was accessible to researchers. Study results: The results of the participants include both positive and negative outcomes. Reflections/critical perspective: There were no relevant issues with public involvement.

## Guidelines and Standards Statement

This manuscript was drafted against STROBE for observational studies.

## Appendix A

**Table A1:** Descriptive data for the quantitative variables.

	N	Minimum	Maximum	Mean	Standard Deviation
SPPB Balance	194	0	4	1.81	1.52
SPPB Speed	194	0	4	1.57	1.33
SPPB GetUp	194	0	10	0.86	1.28
SPPB Total	194	0	12	4.17	3.58
Barthel	194	0	100	58.61	32.97
Lawton and Brody	194	0	8	1.49	2.33
Downton	194	0	7	2.67	1.57

SPPB: Short Physical Performance Battery.
